# TGIF-DB: terse genomics interface for developing botany

**DOI:** 10.1186/s13104-021-05599-4

**Published:** 2021-05-13

**Authors:** Daisuke Tsugama, Tetsuo Takano

**Affiliations:** Asian Natural Environmental Science Center (ANESC), The University of Tokyo, 1-1-1 Midori-cho, Nishi-tokyo-shi, Tokyo, 188-0002 Japan

**Keywords:** Arabidopsis, Garden asparagus, Chickpea, Genomics, Pearl millet, Plant, Rice, Tartary buckwheat

## Abstract

**Objectives:**

Pearl millet (*Pennisetum glaucum*) is a staple cereal crop for semi-arid regions. Its whole genome sequence and deduced putative gene sequences are available. However, the functions of many pearl millet genes are unknown. Situations are similar for other crop species such as garden asparagus (*Asparagus officinalis*), chickpea (*Cicer arietinum*) and Tartary buckwheat (*Fagopyrum tataricum*). The objective of the data presented here was to improve functional annotations of genes of pearl millet, garden asparagus, chickpea and Tartary buckwheat with gene annotations of model plants, to systematically provide such annotations as well as their sequences on a website, and thereby to promote genomics for those crops.

**Data description:**

Sequences of genomes and transcripts of pearl millet, garden asparagus, chickpea and Tartary buckwheat were downloaded from a public database. These transcripts were associated with functional annotations of their *Arabidopsis thaliana* and rice (*Oryza sativa*) counterparts identified by BLASTX. Conserved domains in protein sequences of those species were identified by the HMMER scan with the Pfam database. The resulting data was deposited in the figshare repository and can be browsed on the Terse Genomics Interface for Developing Botany (TGIF-DB) website (http://webpark2116.sakura.ne.jp/rlgpr/).

## Objective

Pearl millet (*Pennisetum glaucum*) is a staple cereal crop for semi-arid regions. Its whole genome was sequenced and putative gene sequences were deduced [1]. Functions of some of the pearl millet genes have also been either examined by experiments or predicted on the basis of their homologies to specific, targeted gene sets with known functions ([2, 3]; for example). However, functional annotations of the pearl millet genes are neither sufficient nor systematic. Situations are similar in many other plant species such as garden asparagus (*Asparagus officinalis*), chickpea (*Cicer arietinum*) and Tartary buckwheat (*Fagopyrum tataricum*) [4–6 respectively, for analyses of their genomes]. *Arabidopsis thaliana* and rice (*Oryza sativa*) are dicot and monocot model species, respectively, and have better functional annotations for each gene ([7, 8]; for example). The objective of the data presented here was to improve the functional annotations of genes of pearl millet by systematic homology searches with databases for Arabidopsis genes, rice genes and protein conserved domains, to develop a platform for browsing the resulting data, and thereby to promote pearl millet genomics.

## Data description

The whole genome sequences and transcript (or protein coding) sequences that were deduced from the genome sequences of pearl millet, garden asparagus, chickpea and Tartary buckwheat as well as genome annotation files in the general feature format (GFF) were downloaded from the International Pearl Millet Genome Sequencing Consortium (IPMGSC) website [9], the Asparagus Genome Project website [10], the National Center for Biotechnology Information (NCBI) Chickpea Genome website (with Genome ID 2992) [11] and the MBKBASE Tartary Buckwheat Genome Project website [12], respectively. The sequences and functional annotations of Arabidopsis proteins (TAIR10 versions) were downloaded from The Arabidopsis Information Resource (TAIR) website [7], and those of rice (RGAP 7 versions) were downloaded from the Rice Genome Annotation Project (RGAP) website [8]. BLASTX on the BLAST + suite [13] was performed with the transcript sequences of those crop species as queries and with either the Arabidopsis protein sequences or rice protein sequences as the database. The threshold E-value was set as 1e − 20, which is more stringent than the default value (10.0), for this analysis. The transcripts (or genes) of the crop species were then associated with the functional annotations of the corresponding Arabidopsis and rice proteins identified by the BLASTX search. Protein sequences of pearl millet, garden asparagus, chickpea and Tartary buckwheat were deduced from their transcript sequences, and the Pfam database [14] was searched by the hmmscan program for HMMER (version 3.3) [15] to identify conserved domains in those proteins. The threshold E-value was set as 1e − 5, which is more stringent than the default value (10), for this analysis. A genomic locus sequence, which consists of exons and introns, and a promoter sequence, which is a 3-kb upstream sequence from the start codon, for each gene were extracted on the basis of the whole genome sequences and the GFF files. The resulting data for the gene sequences and their functional annotations for pearl millet, garden asparagus, chickpea and Tartary buckwheat were deposited in the figshare repository (Data sets 1–34 in Table 1) [16]. A website, Terse Genomics Interface for Developing Botany (TGIF-DB), was developed to browse these data [17] (see Data file 1 in Table 1 for a TGIF-DB interface). The programs in the BLAST+ suite [13] and the genome browser JBrowse [18] were included as a part of TGIF-DB.

**Table 1:** Overview of data files/data sets

Label	Name of data file/data set	File types (file extension)	Data repository and identifier (DOI or accession number)
Data set 1	Ao_gene_seqs.fa	Fasta (.fa) file	Figshare (https://doi.org/10.6084/m9.figshare.13565168.v2) [16]
Data set 2	Ao_promoter_seqs.fa	Fasta (.fa) file	Figshare (https://doi.org/10.6084/m9.figshare.13565168.v2) [16]
Data set 3	Ao_protein_seqs.fa	Fasta (.fa) file	Figshare (https://doi.org/10.6084/m9.figshare.13565168.v2) [16]
Data set 4	Ao_proteins_At_proteins_blastp.txt	Text (.txt) file	Figshare (https://doi.org/10.6084/m9.figshare.13565168.v2) [16]
Data set 5	Ao_proteins_ELM.txt	Text (.txt) file	Figshare (https://doi.org/10.6084/m9.figshare.13565168.v2) [16]
Data set 6	Ao_proteins_Os_proteins_blastp.txt	Text (.txt) file	Figshare (https://doi.org/10.6084/m9.figshare.13565168.v2) [16]
Data set 7	Ao_proteins_Pfam.txt	Text (.txt) file	Figshare (https://doi.org/10.6084/m9.figshare.13565168.v2) [16]
Data set 8	Ao_transcript_seqs.fa	Fasta (.fa) file	Figshare (https://doi.org/10.6084/m9.figshare.13565168.v2) [16]
Data set 9	Ao_transcripts_hairpin_miRNA_blastn.txt	Text (.txt) file	Figshare (https://doi.org/10.6084/m9.figshare.13565168.v2) [16]
Data set 10	Ca_gene_seqs.fa	Fasta (.fa) file	Figshare (https://doi.org/10.6084/m9.figshare.13565168.v2) [16]
Data set 11	Ca_promoter_seqs.fa	Fasta (.fa) file	Figshare (https://doi.org/10.6084/m9.figshare.13565168.v2) [16]
Data set 12	Ca_protein_seqs.fa	Fasta (.fa) file	Figshare (https://doi.org/10.6084/m9.figshare.13565168.v2) [16]
Data set 13	Ca_proteins_At_proteins_blastp.txt	Text (.txt) file	Figshare (https://doi.org/10.6084/m9.figshare.13565168.v2) [16]
Data set 14	Ca_proteins_ELM.txt	Text (.txt) file	Figshare (https://doi.org/10.6084/m9.figshare.13565168.v2) [16]
Data set 15	Ca_proteins_Os_proteins_blastp.txt	Text (.txt) file	Figshare (https://doi.org/10.6084/m9.figshare.13565168.v2) [16]
Data set 16	Ca_proteins_Pfam.txt	Text (.txt) file	Figshare (https://doi.org/10.6084/m9.figshare.13565168.v2) [16]
Data set 17	Ca_transcript_seqs.fa	Fasta (.fa) file	Figshare (https://doi.org/10.6084/m9.figshare.13565168.v2) [16]
Data set 18	Ft_gene_seqs.fa	Fasta (.fa) file	Figshare (https://doi.org/10.6084/m9.figshare.13565168.v2) [16]
Data set 19	Ft_promoter_seqs.fa	Fasta (.fa) file	Figshare (https://doi.org/10.6084/m9.figshare.13565168.v2) [16]
Data set 20	Ft_protein_seqs.fa	Fasta (.fa) file	Figshare (https://doi.org/10.6084/m9.figshare.13565168.v2) [16]
Data set 21	Ft_proteins_At_proteins_blastp.txt	Text (.txt) file	Figshare (https://doi.org/10.6084/m9.figshare.13565168.v2) [16]
Data set 22	Ft_cds_seqs.fa	Fasta (.fa) file	Figshare (https://doi.org/10.6084/m9.figshare.13565168.v2) [16]
Data set 23	Ft_proteins_Os_proteins_blastp.txt	Text (.txt) file	Figshare (https://doi.org/10.6084/m9.figshare.13565168.v2) [16]
Data set 24	Ft_proteins_Pfam.txt	Text (.txt) file	Figshare (https://doi.org/10.6084/m9.figshare.13565168.v2) [16]
Data set 25	Ft_transcript_seqs.fa	Fasta (.fa) file	Figshare (https://doi.org/10.6084/m9.figshare.13565168.v2) [16]
Data set 26	Pg_gene_seqs.fa	Fasta (.fa) file	Figshare (https://doi.org/10.6084/m9.figshare.13565168.v2) [16]
Data set 27	Pg_promoter_seqs.fa	Fasta (.fa) file	Figshare (https://doi.org/10.6084/m9.figshare.13565168.v2) [16]
Data set 28	Pg_protein_seqs.fa	Fasta (.fa) file	Figshare (https://doi.org/10.6084/m9.figshare.13565168.v2) [16]
Data set 29	Pg_proteins_At_proteins_blastp.txt	Text (.txt) file	Figshare (https://doi.org/10.6084/m9.figshare.13565168.v2) [16]
Data set 30	Pg_proteins_ELM.txt	Text (.txt) file	Figshare (https://doi.org/10.6084/m9.figshare.13565168.v2) [16]
Data set 31	Pg_proteins_Os_proteins_blastp.txt	Text (.txt) file	Figshare (https://doi.org/10.6084/m9.figshare.13565168.v2) [16]
Data set 32	Pg_proteins_Pfam.txt	Text (.txt) file	Figshare (https://doi.org/10.6084/m9.figshare.13565168.v2) [16]
Data set 33	Pg_transcript_seqs.fa	Fasta (.fa) file	Figshare (https://doi.org/10.6084/m9.figshare.13565168.v2) [16]
Data set 34	Pg_transcripts_hairpin_miRNA_blastn.txt	Text (.txt) file	Figshare (https://doi.org/10.6084/m9.figshare.13565168.v2) [16]
Data file 1	Figure_TGIF-DB_snapshots.tif	Figure (.tif) file	Figshare (https://doi.org/10.6084/m9.figshare.13565168.v2) [16]

## Limitations

- Some proteins of the species used do not appear to have conserved domains and/or any close homolog in either* Arabidopsis* or rice.
- Some pearl millet genes have been characterized by targeted analyses ([2,3]; for example) but information about such analyses has not been included in the data set presented.

## Data Availability

The datasets generated during and/or analysed during the current study are available in the figshare repository, https://doi.org/10.6084/m9.figshare.13565168.v2 [16]. The data can be browsed on the TGIF-DB website, http://webpark2116.sakura.ne.jp/rlgpr/ [17]. Please see Table 1 and references [10, 11] for details and links to the data.

## Abbreviation

GFF: General feature format

## References

[1] Varshney RK, Shi C, Thudi M, Mariac C, Wallace J, Qi P (2017). Pearl millet genome sequence provides a resource to improve agronomic traits in arid environments. Nat Biotechnol.

[2] Shinde H, Dudhate A, Tsugama D, Gupta SK, Liu S, Takano T (2019). Pearl millet stress-responsive NAC transcription factor PgNAC21 enhances salinity stress tolerance in *Arabidopsis*. Plant Physiol Biochem.

[3] Chanwala J, Satpati S, Dixit A, Parida A, Giri MK, Dey N (2020). Genome-wide identification and expression analysis of WRKY transcription factors in pearl millet (*Pennisetum glaucum*) under dehydration and salinity stress. BMC Genomics.

[4] Harkess A, Zhou J, Xu C, Bowers JE, Van der Hulst R, Ayyampalayam S (2017). The asparagus genome sheds light on the origin and evolution of a young Y chromosome. Nat Commun.

[5] Varshney RK, Song C, Saxena RK, Azam S, Yu S, Sharpe AG (2013). Draft genome sequence of chickpea (*Cicer arietinum*) provides a resource for trait improvement. Nat Biotechnol.

[6] Zhang L, Li X, Ma B, Gao Q, Du H, Han Y (2017). The Tartary buckwheat genome provides insights into rutin biosynthesis and abiotic stress tolerance. Mol Plant.

[7] Berardini TZ, Reiser L, Li D, Mezheritsky Y, Muller R, Strait E, Huala E (2015). The Arabidopsis information resource: making and mining the “gold standard” annotated reference plant genome. Genesis.

[8] Kawahara Y, de la Bastide M, Hamilton JP, Kanamori H, McCombie WR, Ouyang S (2013). Improvement of the *Oryza sativa* Nipponbare reference genome using next generation sequence and optical map data. Rice.

[9] Varshney RK, Shi C, Thudi M, Mariac C, Wallace J, Qi P, et al. International pearl millet genome sequencing consortium (IPMGSC). 2021. http://cegsb.icrisat.org/ipmgsc. Accessed 14 Jan 2021.

[10] Asparagus Genome Project. 2021. http://asparagus.uga.edu/tripal. Accessed 16 Mar 2021.

[11] NCBI genome for *Cicer arietinum* (chickpea). 2021. https://www.ncbi.nlm.nih.gov/genome/2992. Accessed 16 Mar 2021.

[12] MBKBASE, introduction to tartary buckwheat genome project. 2021. http://mbkbase.org/Pinku1. Accessed 16 Mar 2021.

[13] Camacho C, Coulouris G, Avagyan V, Ma N, Papadopoulos J, Bealer K, Madden TL (2009). BLAST+: architecture and applications. BMC Bioinform.

[14] El-Gebali S, Mistry J, Bateman A, Eddy SR, Luciani A, Potter SC (2019). The Pfam protein families database in 2019. Nucleic Acids Res.

[15] Mistry J, Finn RD, Eddy SR, Bateman A, Punta M (2013). Challenges in homology search: HMMER3 and convergent evolution of coiled-coil regions. Nucleic Acids Res.

[16] Tsugama D. TGIF-DB datasets. figshare. 2021. 10.6084/m9.figshare.13565168.v5.

[17] Tsugama D. TGIF-DB. http://webpark2116.sakura.ne.jp/rlgpr. Accessed 14 Jan 2021.

[18] Buels R, Yao E, Diesh CM, Hayes RD, Munoz-Torres M, Helt G (2016). JBrowse: a dynamic web platform for genome visualization and analysis. Genome Biol.

